# Status report of the first AMS laboratory in the Czech Republic at the Nuclear Physics Institute, Řež

**DOI:** 10.1007/s11696-023-02904-2

**Published:** 2023-06-14

**Authors:** Jan Kučera, Kateřina Pachnerová Brabcová, Mojmír Němec, Jan Kameník, Jakub Trubač, Veronika Brychová, Ivo Světlík, Jan John, Roman Garba, Martin Daňo

**Affiliations:** 1 Czech Academy of Sciences, Nuclear Physics Institute, Řež 130, 250 68 Husinec–Řež, Czech Republic; 2 Faculty of Nuclear Sciences and Physical Engineering, Czech Technical University in Prague, Břehová 7, 115 19 Prague 1, Czech Republic; 3 Czech Academy of Sciences, Institute of Archaeology, Prague, Letenská 123/4, 118 01 Prague 1, Czech Republic

**Keywords:** Accelerator mass spectrometry, ^10^Be, ^14^C, ^26^Al, ^129^I, ^236^U, Radiocarbon dating

## Abstract

The first accelerator mass spectrometry (AMS) laboratory in the Czech Republic has been established and put into routine operation in February 2022. Here we briefly describe the facilities available, namely a 300 kV multi-isotope low-energy AMS system (MILEA) capable of determination ^10^Be, ^14^C, ^26^Al, ^41^Ca, ^129^I, isotopes of U, especially ^236^U, Pu and other actinoids, and accessories for ^14^C measurements, which include a gas interface system, a preparative gas chromatography system for compound-specific radiocarbon dating analysis, and an isotope-ratio mass spectrometer. The first results achieved for separation and measurement of the above radionuclides (except for ^41^Ca) are also reported, with the main focus on ^14^C measurements. A specimen breakdown of 729 graphitised samples analysed for ^14^C so far is presented, as well as a proof of measurement stability of the MILEA system obtained by analysis of radiocarbon standards and analytical blanks. For the other radionuclides, well proven or novel procedures for sample preparation and measurement are presented.

## Introduction

With regard to increasing demands for low-level measurement of long-lived radionuclides, the first AMS laboratory in the Czech Republic has recently been established within a consortium of the Nuclear Physics Institute (NPI) of the Czech Academy of Sciences (CAS), the Faculty of Nuclear Sciences and Physical Engineering of the Czech Technical University in Prague and the Archaeological Institute in Prague of CAS. The laboratory has been built in new premises of NPI, Řež. It is equipped with a 300 kV multi-isotope low-energy AMS system (MILEA) developed in a collaboration of Ionplus AG and ETH Zurich, Switzerland. Scientific progress allows for new types of ion sources, accelerators and detectors, which result in the design and production of compact and economic AMS systems, like MILEA. Thus, AMS systems with a low terminal voltage in the sub-500 kV range have recently become available (Synal et al. 2007; Christl et al. 2013; Zondervan et al. 2015; Maxeiner et al. 2019; Gautschi et al. 2021; Scognamiglio et al. 2021; Fujita et al. 2021; Saito-Kokubu et al 2021). Although the majority of such AMS systems are dedicated ^14^C facilities, which are capable of providing high measurement efficiency, stability and reproducibility even at accelerator voltages down to 50 kV (Synal 2020), an increasing number of low-voltage AMS systems were constructed in recent years, which retain the multi-isotope capability. This is also the case of MILEA with its 300 kV terminal voltage capable of, but not limited to, determination of ^10^Be, ^14^C, ^26^Al, ^41^Ca, ^129^I, isotopes of U, Pu and other actinoids with detection limits competitive with those obtained at AMS systems with a higher terminal voltage. This device thus appears optimal for a Czech national project “Ultra-trace isotope research in social and environmental studies using accelerator mass spectrometry”, acronym RAMSES. MILEA is composed, as the majority of AMS systems, from five basic parts: (i) an ion source with a sample changer; (ii) a low-energy (LE) part with an injector of negatively charged ions; (iii) a tandem accelerator with a stripper, where the ion beam of negative ions is transformed to positively charges ions; (iv) a high energy (HE) part for analysis of accelerated positive ions; (v) a detector for measurement of rare isotopes. A description and MILEA performance has already been given elsewhere (Gautschi et al. 2021; Kučera et al. 2022). Therefore, the aim of this paper is to present only the most important features of this AMS system together with information on MILEA accessories newly acquired. Also, the first results achieved till November 2022 are briefly outlined, which concern measurements of the above listed radionuclides (except for ^41^Ca, which is presently out of our focus), as well as well-known or newly developed sample preparation procedures tested.

## Experimental

### MILEA system and its accessories

The ion source is the well-proven MICADAS type (Synal et al. 2007) employing sputtering of a sample pressed in a sample holder (cathode) with accelerated and focused Cs ions. The sample changer uses a 40-position magazine allowing measurement without interruption, because it can be exchanged without losing vacuum.

The negative ion beam enters the accelerator with the ion energy of tens kV and is accelerated up to the terminal voltage. During passage through the accelerator the ion beam enters a collision cell filled with He gas having an areal density (gas thickness) of a few µg cm^−2^, where negative ions become positively charged, and simultaneously molecular ions are destroyed. The vacuum insulated accelerator uses the terminal voltage tuneable up to 300 kV and takes advantage of the use of He as the stripping gas, which accomplishes a higher ionizing efficiency and a smaller ion beam scatter compared with other gases, such as Ar, O_2_ and N_2_ (Schultze-König et al. 2011, Vockenhuber et al. 2013).

**Fig. 1:**
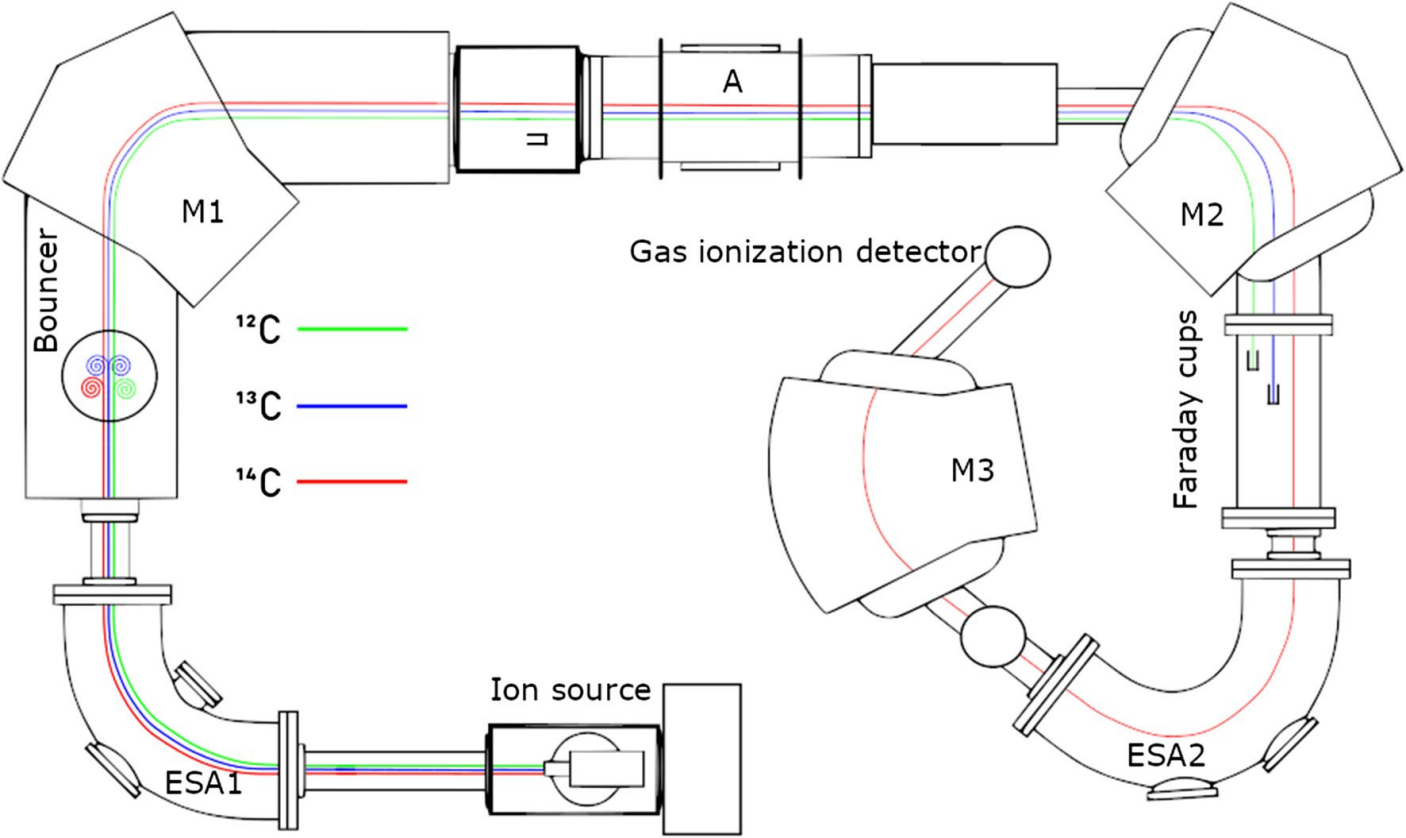
Layout of MILEA: A—accelerator, ESA1 and ESA2—low-energy electrostatic analyser and high-energy electrostatic analyser, respectively, *M*1—low-energy magnet, *M*2 and *M*3—high-energy magnets

A 75-nm thick Si_3_N_4_ degrader foil is inserted in the HE analyser for ^10^Be measurement to help to eliminate ^10^B isobaric interference. MILEA uses as the end-of-line detector a gas ionization detector with two collective electrodes, which is filled with isobutane. For ^26^Al^2+^ measurement, an absorption cell filled with isobutane is placed in front of the detector to remove the intense *m*/*z* ambiguity with ^13^C^+^ ions. An important construction innovation is the achromatic arrangement of all parts of the ion optics, which guarantees a link-up of the focal points of the individual parts of the ion optics even for various ion mass/energy ratios. A simplified layout of MILEA with paths of ^12^C, ^13^C, and ^14^C ions is depicted in Fig. 1.

### Accessories

#### Gas interface system (GIS)

For ^14^C analysis, our MILEA system is also equipped with GIS (Ionplus AG, Switzerland), which allows for direct measurement of gaseous ^14^CO_2_ instead of the determination ^14^C in graphitised samples. The GIS is used in three main ways: (i) as a source of CO_2_ prepared directly in a coupled elemental analyser (Elementar, Germany); (ii) using CO_2_ stored in quartz ampoules, which are broken in a GIS ampoule cracker; (iii) using CO_2_ from a gas bottle usually containing normalization standards and blanks. In all cases, a zeolite CO_2_ trap and flushing with helium are used for proper CO_2_ dosing through a capillary directly to the cathode in the caesium sputtering source.

#### Preparative gas chromatography system

A gas chromatography system LECO Pegasus BT2 MS-TOF combined with Agilent 8890 preparative gas chromatograph coupled to a Gerstel preparative fraction collector is used for monitoring of targeted compounds and for compound-specific radiocarbon dating analysis (CSRA). CSRA is powerful, yet challenging tool, used, e.g. for isolation of the most concentrated fatty acids in pottery lipid extracts and subsequent radiocarbon dating to obtain a radiocarbon date of archaeological pottery (Brychová et al. 2022).

#### Isotope-ratio mass spectrometer (IRMS)

Measurement of the relative abundance of stable isotopes is carried out using a Thermo IsoLink Elemental Analyser connected to a Thermo Delta V Advantage (Thermo Fisher Scientific, Bremen, Germany) IRMS in a Continuous Flow IV system. The system allows measurement of stable isotopes of carbon (δ^13^C), nitrogen (δ^15^N) and sulphur (δ^34^S) in a combustion regime. Deuterium (δ^2^H) and oxygen (δ^18^O) isotopes are determined in a pyrolytic regime. Samples are wrapped in tin capsules for the combustion reaction. On the contrary for the pyrolytic analysis samples must be placed in silver capsules. A minimal sample mass depends on the wt% amount of each element. The released gases separated in a gas chromatography column are transferred to the mass spectrometer source through a capillary. Isotope ratios are reported as delta (δ) values and expressed relative to V-PDB or V-SMOW scale. The δ values are normalized to a calibration curve based on international standards.

#### MILEA factory- and on-site acceptance tests (FAT and SAT, respectively)

FAT and SAT were carried out or assisted by Ionplus specialists to determine MILEA basic performance parameters at Ionplus and NPI, respectively. The parameters for the measurement of ^10^Be, ^14^C, ^26^Al, ^129^I, ^233,236^U included the stable isotope transmission from the injector to the HE Faraday cup (FC), the rare isotope transmission from the HE FC to the detector, the rare isotope HE current, the rare/stable isotope blank ratio (in the case of ^236^U the abundance sensitivity), and the single- and overall-sample scatter determined by using well established standards and blanks, as has been described in detail elsewhere (Kučera et al. 2022).

## Results and discussion

### MILEA performance

The FAT and SAT of MILEA were carried out in the time span of almost one year due to Covid-19 restrictions during the assemblage and installation at NPI and showed very good reproducibility (Kučera et al. 2022). For the sake of simplicity, we present in Table 1 only SAT results for one of the most important parameters—blank ratios for rare/stable isotope ratios and compare them with those for other AMS systems. It is obvious that the values for MILEA are competitive even with larger AMS systems (with a higher terminal voltage).

**Table 1:** Blank values for rare/stable isotope ratios achieved in SAT of MILEA and their comparison with values reported for AMS systems with different terminal voltages

AMS system maximal terminal voltage	Isotope pair				
	^10^Be/^9^Be	^14^C/^12^C^a^	^26^Al/^27^Al	^129^I/^127^I	^236^U/^238^U^b^
0.2 MV (Christl et al. 2013)	n.a	4E−15	n.a	n.a	n.a
0.3 MV (Maxeiner et al. 2019)	1.5E−15	n.a	6E−15	1E−13	4E−13
0.3 MV MILEA (Kučera et al. 2022)	< 1.4E−15	8–9E−16	< 2.6E−15	2–7E−14	4.5E−13
0.5 MV (Zondervan et al. 2015)	5E−15	3.4E−16	6E−15	n.a	n.a
0.7 MV (Christl et al. 2013)	3E−15	n.a	n.a	1E−13	n.a
1 MV (Chamizo et al. 2008)	3−4E−14	1E−15	n.a	3–4E−13	n.a
1 MV (Stan-Sion et al. 2014)	2.7E−14	1.8E−15	1.2E−15	1.7E−13	n.a
1 MV (Bhushan et al. 2019)	5.8E−16	3.9E−16	1.6E−14	n.a	n.a
3 MV (Kieser et al. 2015)	6.7E−15	8.8E−16	4.1E−15	1.8E−14	n.a

^a^Graphite samples

^b^Abundance sensitivity

There is no stable isotope of U; therefore, measurement of the ^236^U/^238^U ratio was employed. There is also no real blank material for measurement of the ^236^U/^238^U ratio due to overall ^236^U contamination of the environment by the global fallout. Therefore, instead of the blank ratio, the abundance sensitivity can only be measured using, e. g. Vienna—KkU material recognized as kind of a primary standard (Steier et al. 2008). It can be mentioned that the blank ratio for the ^14^C/^12^C pair was also measured for gaseous samples and somewhat worse value of < 2.0E−14 was obtained compared to that for graphite samples in agreement with results from another laboratory (Mollenhauer et al. 2021). However, the former measurement mode has other advantages. Direct measurements of ^14^CO_2_ can be performed on ultra-small samples containing only 3–100 μg of carbon with the GIS + MILEA coupling. The gas measurement is the ideal solution not only for small samples but also for all specimens for which a lower precision is acceptable, such as for screening and high throughput studies.

### Measurement of ^14^C

**Fig. 2:**
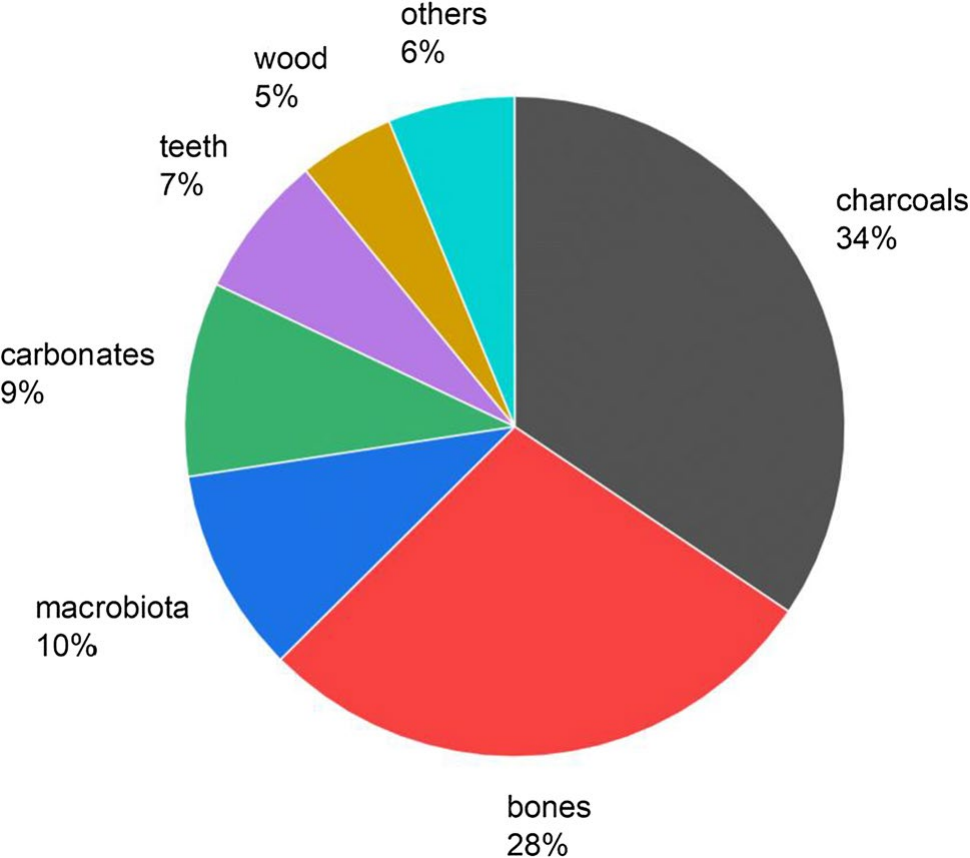
Breakdown of specimen types analysed in NPI radiocarbon laboratory in the period February–November 2022

During February–November 2022, 729 graphite samples were analysed. The samples measured consisted mostly of charcoals, bones and teeth, but also wood, microbiotas and carbonates, each of them requiring specific sample preparation procedures. The specimen breakdown is presented in Fig. 2.

The range of specimens demonstrates our preparedness to employ ^14^C measurements for various archaeological and environmental studies.

**Fig. 3:**
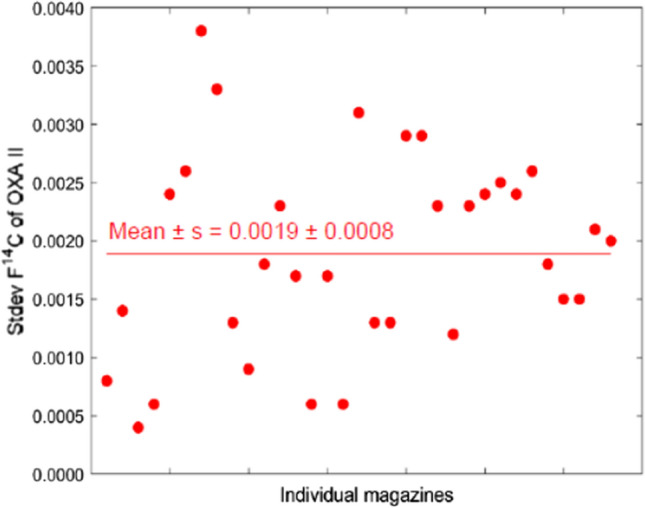
F^14^C standard deviations of OXA II radiocarbon standards in individual magazines (points) and seven months average (line)

**Fig. 4:**
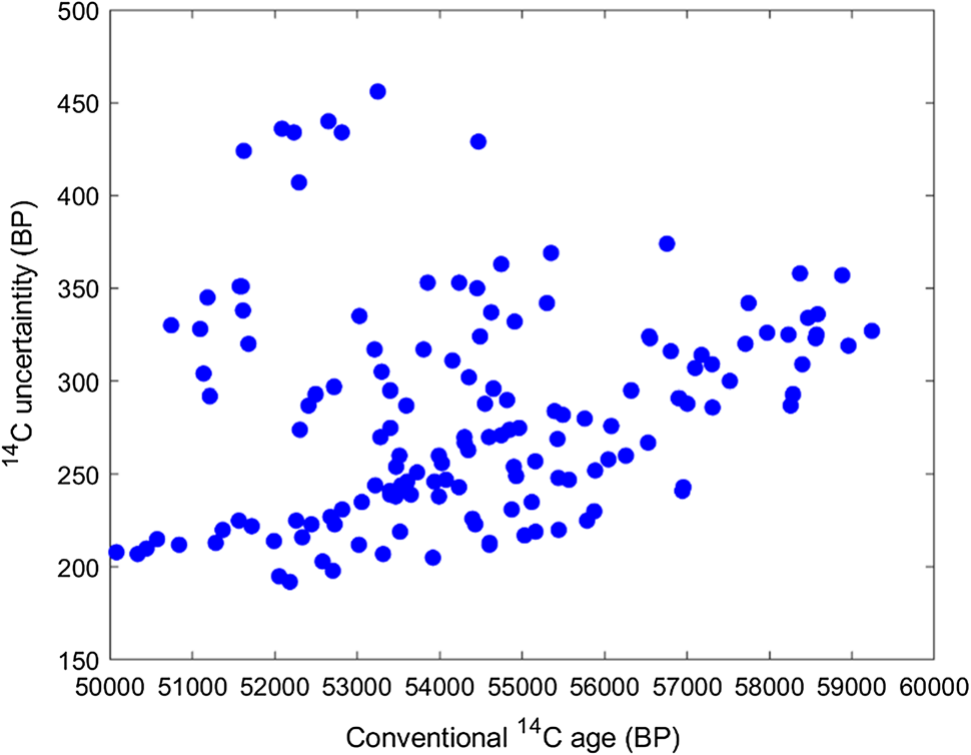
Scatter of blank uncertainties as a function of the conventional radiocarbon age in years before present (BP) measured in the period February-August 2022 for all PHA blanks

Together with each set of samples in individual magazines, 3–5 pairs of radiocarbon standards (OXA II) and analytical blanks (Phthalic anhydride—PHA) were analysed. This experimental set-up allowed us to test the stability of our MILEA system. The results obtained illustrating the favourable stability are depicted in Figs. 3 and 4.

### Measurement of ^10^Be and ^26^Al

For measurement of cosmogenic radionuclides ^10^Be and ^26^Al the performance of two separation procedures for the preparation Be and Al samples for AMS measurements was monitored using a radioactive ^7^Be tracer (*T*_1/2_ = 53.2 d) and instrumental neutron activation analysis (Řanda et al. 2007) to determine Al. One procedure tested employed separation using of two extraction chromatography resins (Binnie et al. 2015), the other procedure employed a combination of precipitation and ion exchange chromatography (Ruszkiczay-Rüdiger et al. 2021). Both monitoring methods were found efficient and suitable in the development, calibration, and control of chemical separation processes for Al and Be-related AMS measurements (Kameník et al. 2023a). To study burial dating of an Early Pleistocene hominin site at Korolevo, Transcarpathia, western Ukraine, the physical and chemical sample treatment purity control was performed in co-operation with Helmholtz Zentrum Dresden-Rossendorf (HZDR) at the Dresden AMS (DREAMS) facility. The cobbles (massive quartz, quartzite and sandstone) from a buried fluvial terrace rich in stone artefacts and quartz pebbles of modern Tisza riverbed were selected for analysis. To examine potential effects of inhomogeneous sample composition or incomplete chemical separation on the determination of ^10^Be and ^26^Al, the follow up mass scans were used on the LE magnet after the routine AMS measurement to evaluate the effectiveness of the physical and chemical sample treatment and their possible impact on the AMS analysis results. For instance, it has been found that the ^10^Be current reduction in AMS measurement was a function of the BeO/TiO_2_ molar ratio (Kameník et al. 2023b).

### Sample processing and measurement of ^129^I

The ^129^I nuclide is determined mainly in environmental samples, such as soil, vegetation, sea water, milk, air, etc. (Suzuki et al. 2007; Hou et al. 2013; Jabbar et al. 2013; Zhang et al. 2021). Processing ^129^I samples is closely related to their nature and the required final form suitable for measurement, which is mostly AgI. The main issue in sample processing for ultra-trace analysis is the volatility of iodine and some of its compounds and therefore the resulting risk of significant analyte loss. Another risk is the contamination coming from chemicals or (radio)tracers, which leads to incorrect results and the need for accurate determination of a blank sample.

A separation method is currently being developed that would be simple, without the use of complex instrumentation, and providing also high chemical yields. The idea is to oxidize the whole sample in aqueous solutions of K_2_S_2_O_8_ at slightly elevated temperature to degrade organic compounds, and also to stabilize iodine in the IO_3_^−^ form and thus minimize its losses. The results of these measurements showed consistent values of ^129^I/^127^I at the level of 2 × 10^–13^, and Woodward iodine blank sample (Delmore et al. 2010) showed good agreement of isotopic ratios with the results obtained in the acceptance tests (Kučera et al. 2022).

The first measurements of ^129^I was carried out on the MILEA device in order to gain practical experience. Standards of ^129^I and in-house background samples were measured. The in-house background samples were prepared by simple precipitation of common laboratory iodine-containing chemicals, KI and CsI with the AgNO_3_. The results of these measurements showed a good agreement of the isotopic ^129^I/^127^I ratios with the results obtained in the acceptance tests.

### Measurement of actinoids

Research has mostly been focused on development of new procedures of ^236^U determination to be able to use suitable properties of this radionuclide for a number of applications, such as overall monitoring of environmental radioactive contamination, for nuclear safeguards and nuclear forensics purposes, tracing of various natural processes, e.g. ocean currents, soil erosion and others. A new fluoride target matrix preparation method was proposed and tested in co-operation with Vienna Environmental Research Accelerator (VERA) AMS facility, which aimed especially at determination of trace amounts of ^236^U in environmental samples. Overall UF_5_^−^ ion yields as high as 4.89% were achieved during the sputtering using non-isotopic carriers (Ca and Nd) in the form of CaF_2_ or NdF_3_ in the combination with PbF_2_. This is about 20-times higher value than that achieved for the reference oxidic matrix during substantially shorter sputtering time (Prášek et al. 2020). This procedure has further been modified in co-operation with Laboratory of Ion Beam Physics, ETH Zurich and VERA AMS Laboratory, University Vienna (Prášek et al. 2022) and extended for the preparation of a target sample material for ^239^Pu (*T*_1/2_ = 2.41E+4 y) measurement with MILEA AMS (Fenclová et al. 2022).

## Conclusions

The factory- and on-site acceptance tests for MILEA AMS system achieved at Ionplus AG and NPI, respectively, showed that the parameters for measurement of ^10^Be, ^26^Al, ^129^I, and actinoids are competitive with larger AMS systems. The MILEA performance tested in the time span of almost one year (due to a delay in assemblage and installation caused by the COVID-19 restrictions) also yielded very good reproducibility, which was further confirmed by follow up analyses of ^14^C standards and blanks as reported in this work. Since the assay of ^14^C is planned to occupy the major part of MILEA measurement time, the system has also been equipped with auxiliary devices allowing analysis of gaseous samples using GIS, the gas chromatograph for CSRA and IRMS for measurement of δ^2^H, δ^13^C, δ^15^N, δ^18^O and δ^34^S values to complement and extend the applicability of our AMS system for various kinds of ^14^C measurements for different purposes. Thus, the equipment required for all needs of RAMSES project, namely low-level measurement of long-lived radionuclides in archaeological, geological and environmental samples, has been acquired, installed, tested and put into routine operation. On the other hand, measurement of other nuclides, in addition to those mentioned in this study, for various applications is not excluded in the future.

Concerning sample preparation procedures, considerable part of them had to be developed and tested in co-operating laboratories abroad to overcome the COVID-19—induced delay in putting new laboratories into operation. Nevertheless, the developed sample preparation procedures for measurement of ^10^Be, ^26^Al, ^129^I and selected actinoids are now ready for implementation in RAMSES project in the AMS laboratory at Řež. Their further improvements may be realized in the near future, as well as an extension of the scope of our project for other nuclides.
